# Macrophage-Derived Protein S Facilitates Apoptotic Polymorphonuclear Cell Clearance by Resolution Phase Macrophages and Supports Their Reprogramming

**DOI:** 10.3389/fimmu.2018.00358

**Published:** 2018-03-01

**Authors:** Delphine Lumbroso, Soaad Soboh, Avi Maimon, Sagie Schif-Zuck, Amiram Ariel, Tal Burstyn-Cohen

**Affiliations:** ^1^Department of Biology, The Faculty of Natural Sciences, University of Haifa, Haifa, Israel; ^2^Faculty of Dental Medicine, Institute for Dental Sciences, Hadassah Medical School, Hebrew University of Jerusalem, Jerusalem, Israel

**Keywords:** inflammation, macrophages, protein S, apoptosis, efferocytosis

## Abstract

The complete resolution of inflammation requires the uptake of apoptotic polymorphonuclear cells (PMN) by local macrophages (efferocytosis) and the consequent reprogramming of the engulfing phagocytes to reparative and pro-resolving phenotypes. The tyrosine kinase receptors TYRO3, AXL, and MERTK (collectively named TAM) are fundamental mediators in regulating inflammatory responses and efferocytosis. Protein S (PROS1) is a ligand for all TAM receptors that mediates various aspects of their activity. However, the involvement of PROS1 in the resolution of inflammation is incompletely understood. Here, we report the upregulation of *Pros1* in macrophages during the resolution of inflammation. Selective knockout of *Pros1* in the myeloid lineage significantly downregulated macrophage pro-resolving properties. Hence, *Pros1*-deficient macrophages engulfed fewer apoptotic PMN remnants *in vivo*, and exogenous PROS1 rescued impaired efferocytosis *ex vivo*. Moreover, *Pros1*-deficient peritoneal macrophages secreted higher levels of the pro-inflammatory mediators TNFα and CCL3, while they secreted lower levels of the reparative/anti-inflammatory IL-10 following exposure to lipopolysaccharide in comparison to their WT counterparts. Moreover, *Pros1*-deficient macrophages expressed less of the anti-inflammatory/pro-resolving enzymes arginase-1 and 12/15-lipoxygenase and produced less of the specialized pro-resolving mediator resolvin D1. Altogether, our results suggest that macrophage-derived PROS1 is an important effector molecule in regulating the efferocytosis, maturation, and reprogramming of resolution phase macrophages, and imply that PROS1 could provide a new therapeutic target for inflammatory and fibrotic disorders.

## Introduction

Inflammation normally resolves in an active process that eliminates the inflammatory effector components that harm the host (1–4). This is hallmarked by leukocyte apoptosis and clearance by macrophages (5–8). Apoptotic cell (AC) engulfment by phagocytes is mediated by signals that are expressed on the surface of ACs and their corresponding receptors [reviewed in Refs. (7, 9)]. Efferocytosis leads to macrophage reprogramming/immune-silencing (5, 10–13) in response to bacterial moieties through specific kinases, such as p38 MAPK, JNK, and cAMP production (14, 15). Macrophage reprogramming is defined by a reduction in the release of pro-inflammatory cytokines and chemokines, concomitant with the production of TGFβ and IL-10 (16–18), cytokines that can promote resolution and wound repair. In addition, the uptake of ACs promotes the expression of 15-lipoxygenase (LO)-1, which is involved in the generation of pro-resolving lipid mediators by macrophages (19–21). A new resolution phase macrophage phenotype—distinguishable from either M1 or M2—and characterized by low expression of CD11b, is generated from the CD11b^high^ phenotype upon the engulfment of threshold numbers of apoptotic polymorphonuclear cells (PMN) (21). This phenotypic conversion of macrophages results in significant immune-silencing in addition to the reduction in the expression of arginase-1, surface CD11b, and F4/80 (21). Specifically, CD11b^low^ macrophages stop producing TNFα and IL-1β, but increase the production of TGFβ and the expression of 12/15-LO, and emigrate to the lymphatics (21).

Protein S (PROS1) is a secreted multifunctional glycoprotein encoded by the *Pros1* gene and best known for its potent anticoagulant activity as a cofactor for activated protein C (22). Outside the coagulation system, PROS1 functions as an agonist for the TAM family of receptor tyrosine kinases, comprising TYRO3, AXL, and MERTK (23, 24), which are negative regulators of immunity (25–28). TAM receptors and their cognate ligands PROS1 and growth-arrest-specific-6 (GAS6) are expressed by immune cells, including macrophages and dendritic cells (DCs) (29, 30). This signaling axis dampens immune reactivity and contributes toward resolving inflammation through at least two distinct mechanisms: the molecular inhibition of the production and the secretion of pro-inflammatory cytokines and by the phagocytic clearance of ACs.

In macrophages, MERTK inhibits the production and secretion of the pro-inflammatory cytokine TNFα following lipopolysaccharide (LPS) exposure (31), while unchallenged macrophages isolated from TAM triple-mutant mice express aberrantly high levels of MHC class II molecules and IL-12 (32). The role of PROS1 as a TAM agonist *in vivo* was demonstrated in the phagocytic clearance of photoreceptor outer segments by retinal-pigment epithelial cells (33) and in T-cell–DC immune interaction (29). However, its role in macrophage-mediated resolution of inflammation has not been investigated.

To test the physiological role of PROS1 in resolution phase macrophages, we generated mice genetically ablated for *Pros1* expression in the myeloid lineage and assessed macrophage efferocytosis and reprogramming in a model of zymosan A-induced peritonitis. Here, we report that *Pros1* is produced by resolution phase macrophages and promotes key features of these macrophages. We show that *in vivo* efferocytosis is compromised in PROS1-deficient macrophages. Exogenous PROS1 was able to rescue the engulfment of ACs *ex vivo*. We also show that PROS1 ablation attenuates macrophage conversion toward anti-inflammatory/reparative phenotypes, as determined by their cytokine secretion balance. Moreover, PROS1 deficiency resulted in the hampered expression of the pro-resolving enzymes arginase-1 and 12/15-LO, as well as the latter’s product, resolvin D1 (RvD1). Hence, PROS1 is a key mediator in successful resolution of inflammation.

## Materials and Methods

### Reagents

ELISA kits for mouse TNFα, IL-10, and IL-6 were obtained from Biolegend; a mouse CCL3 detection kit was obtained from R&D Systems. LPS (from *Escherichia coli*, clone 055:B5) and zymosan A (from *Saccharomyces cerevisiae*) were purchased from Sigma-Aldrich (St. Louis, MI, USA). Docosahexaenoic acid (DHA) was purchased from Cayman Chemicals.

### Murine Peritonitis

Male C57BL/6 LysM*^Cre/+^*; *Pros1^fl/fl^* and *Pros1^fl/fl^* mice (8–10 week old), were injected intra-peritoneally (i.p.) with freshly prepared zymosan A (1 mg/25 g body weight; Sigma-Aldrich) from *S. cerevisiae* in sterile PBS or left unchallenged. After 24, 48, and 66 hrs, mice were euthanized and peritoneal exudates were collected by lavage with 5 ml of sterile PBS. All animal experiments were approved by the Hebrew University—Hadassah ethics committee.

### Macrophages Isolation

Peritoneal exudates were recovered with 5 ml PBS and centrifuged (1300 rpm for 5 min). Then, cells and cell-free peritoneal fluids were separated. Peritoneal macrophages were isolated from exudate cells by EasySep™ mouse PE-positive selection magnetic beads kit (StemCell Technologies) using PE anti-mouse F4/80 antibody (#123110, Biolegend). Isolated macrophages were used for RT-qPCR, microscopic analysis, and *ex vivo* stimulation experiments.

### Quantitative Real-time PCR (qPCR)

Isolated macrophages were harvested, washed once with PBS, and their RNA content was isolated with TRIZOL (Sigma-Aldrich). cDNA was synthesized with qScript cDNA synthesis kit (Quanta Biosciences). Real-time PCR reactions were performed in triplicates using KAPA SYBR FAST qPCR Kit (KAPA-Biosystems) following the manufacturer’s instructions on a CFX96 Real-Time PCR Cycler (Bio-Rad). The reactions were normalized to *mGapdh* using the ΔΔ threshold cycle (Ct) method. Specificity of the primers was confirmed by dissociation curves. Mouse primer sequences were as follows: *mPros1* Forward 5'-TTC CGT GTT GGC TCA TTC C-3'; *mPros1* Reverse 5'-TTG GTC TGA GAT GGC TTT GAC A-3', *mGapdh*-Forward 5'-AGT TGG GAT AGG GCC TCT CTT-3', and *mGapdh*-Reverse 5'-TCC CAC TCT TCC ACC TTC GA-3'.

### *In Vivo* Engulfment Assay

Peritoneal macrophages were isolated at the indicated time points and plated (2 × 10^5^ cells/well) onto eight-well chamber glass slides for 2 h at 37°C in RPMI 1640 (Gibco) to allow adhesion. Then, the cells were rinsed briefly with PBS, fixed for 15 min with 4% paraformaldehyde (PFA) containing 5% sucrose in PBS, and washed twice in PBS. Fixed cells were incubated overnight at 4°C with phalloidin CF488A conjugate (5 units/ml, for F-actin, Biotium). Then, the cells were washed twice with PBS, stained with Hoechst (20 µg/ml for nuclear DNA, Invitrogen H3570) for 5 min, and washed thoroughly, but gently. Mounted slides were kept in the dark at 4°C until analyzed. Macrophages and engulfed apoptotic remnants were enumerated under a confocal fluorescent microscope (Zeiss) as previously described (21). Briefly, macrophages from five to eight fields per chamber (approx. 200 cells) were analyzed per mouse, and the average number of neutrophils engulfed per macrophage, as well as the number of macrophages that have actively engulfed apoptotic moieties, was calculated. Engulfed nuclear material was identified by a spherical bright nuclear Hoechst staining of 1–10 µm in diameter. Then, phagocytic efficiency was calculated as in Ref. (34). Briefly, the phagocytic efficiency index was calculated based on a weighted average of ingested apoptotic DNA-containing particles per macrophage and the number of macrophages containing a certain number of such particles. Phagocytic efficiency (%) = [(1 × *X*_(1)_ + 2 × *X*_(2)_ + 3 × *X*_(3)_ … + *n* × *X*_(_*_n_*_)_)/total number of macrophages] × 100. *X*_(_*_n_*_)_ represents the number of macrophages containing *n* apoptotic particles (*n* = 1, 2, 3, …, up to a maximum of six points for more than five apoptotic particles ingested per macrophage).

### *In Vitro* Engulfment of ACs

Jurkat cells (10 × 10^6^) were induced for apoptosis with staurosporine (Sigma-Aldrich, 1 µM/10^6^ cells in 1 ml RPMI medium containing pen/strep/glutamine and 10% FBS) for 5 h. Cells were washed three times in PBS, labeled with CypHer5E (GE Healthcare; 1 µl CypHer5E/1 ml serum-free medium) for 30 min, and washed twice with PBS. Peritoneal macrophages were isolated as described, and 150 × 10^3^ cells were plated on an eight-well glass chamber slide (Nunc) and fed with 750 × 10^3^ pre-labeled apoptotic Jurkat cells for 4 h in a total of 150-µl medium with or without soluble PROS1 (25 nM; from Enzyme Research Laboratories). Next, the medium was aspirated, and bound cells were washed gently with PBS. Adherent cells were subsequently fixed in 200 µl of 4% PFA; 5% sucrose for 15 min. Cells were washed in PBS and incubated with 200 µl phalloidin (5 U/ml, CF488A conjugate, Biotium) at 4^°^C overnight. Then, cells were washed three times with PBS (10 min each) and stained in 200 µl of Hoechst 3570 (20 µg/ml, Invitrogen) for 5 min, and rinsed with PBS. The chambers were removed, mounted with Fluoromount G, and visualized under a Nikon A1 confocal microscope. The number of CypHer5E^+^ engulfed ACs per macrophage was scored.

### Cytokine and Chemokine Secretion *Ex Vivo*

Peritoneal macrophages were isolated using PE selection magnetic beads (StemCell Technologies) and incubated (5 × 10^6^ cells in 5 ml of culture media) with LPS (1 µg/ml) or vehicle in RPMI 1640 under a humidified 5% CO_2_ atmosphere at 37°C. After 24 h, the supernatants were collected, and their TNFα, IL-6, CCL3, and IL-10 contents were determined by standard ELISA (Biolegend kits for TNFα, IL-6, and IL-10 and R&D Systems kit for CCL3).

### Western Blot Analysis

Protein extracts from equal volumes of peritoneal fluids or equal total protein content of isolated macrophages were subjected to SDS-PAGE using 10% polyacrylamide gels, transferred (1 h, 15 V) to PVDF membranes (Bio-Rad), and blocked for 1 h with 5% BSA in TBST (0.1% Tween 20 in Tris-buffered saline). Then, membranes were immuno-blotted overnight at 4°C with either goat anti-mouse CD11b (M-19, 1:200, SantaCruz Biotechnology), goat anti-mouse arginase-1 (ab60176, 1:20,000, Abcam), rabbit anti-mouse 12/15-LO (160704, 1:1,000, Cayman Chemical), rabbit anti-mouse PROS1 (AB15928, 1:1,000, Merck Millipore), and goat anti-mouse actin (I-19, 1:500, SantaCruz Biotechnology). Then, the membranes were washed three times with TBST and incubated with the appropriate HRP-conjugated secondary antibodies (1:10,000, 1 h, room temperature, Jackson ImmunoResearch). Blots were washed and developed using the EZ-ECL (Biological Industries) chemiluminescence kit and analyzed using the LAS-4000 luminescent image analyzer (Fujifilm) and the TotalLab TL-100 software (Nonlinear Dynamics). Band densitometric intensities among different samples were normalized to actin.

### RvD1 Quantification

Peritoneal macrophages were isolated 66 h post peritonitis initiation from the indicated mice; 10^6^ cells were resuspended in 1-ml medium and immediately supplemented with DHA (20 µM, Cayman Chemicals) for 4 h. Then, the incubation was stopped with cold MeOH, the supernatants were collected in a glass tube, and the MeOH was allowed to evaporate completely; 5 ml of ddH_2_O and 200 µl of MeOH (pH 3.5) were added to the tubes and the samples were loaded through activated Sep-pak Vac 6cc (500 mg) C18 Cartridges (Waters, WAT043395) allowing for MeOH-activated lipid binding. Next, the bound lipids were released using methyl formate, which was then evaporated completely while adding small amounts of MeOH under nitrogen flow. Finally, RvD1 content in lipid-extracted samples was determined using a commercial ELISA kit (Cayman Chemicals), according to the manufacturer’s instructions.

### Statistical Analysis

Experiments were repeated at least three times with at least three replicates per experiment. Results were analyzed by two-way analysis of variance (for multiple groups) or Student’s *t*-test (for comparison between two groups), unless otherwise mentioned. *P*-values (*P*), *P ≤ 0.05, **P ≤ 0.01, and ***P ≤ 0.001, were considered statistically significant. Results are expressed as means ± SEM.

## Results

### PROS1 Expression Is Upregulated in Resolution Phase Macrophages

**Figure 1:**
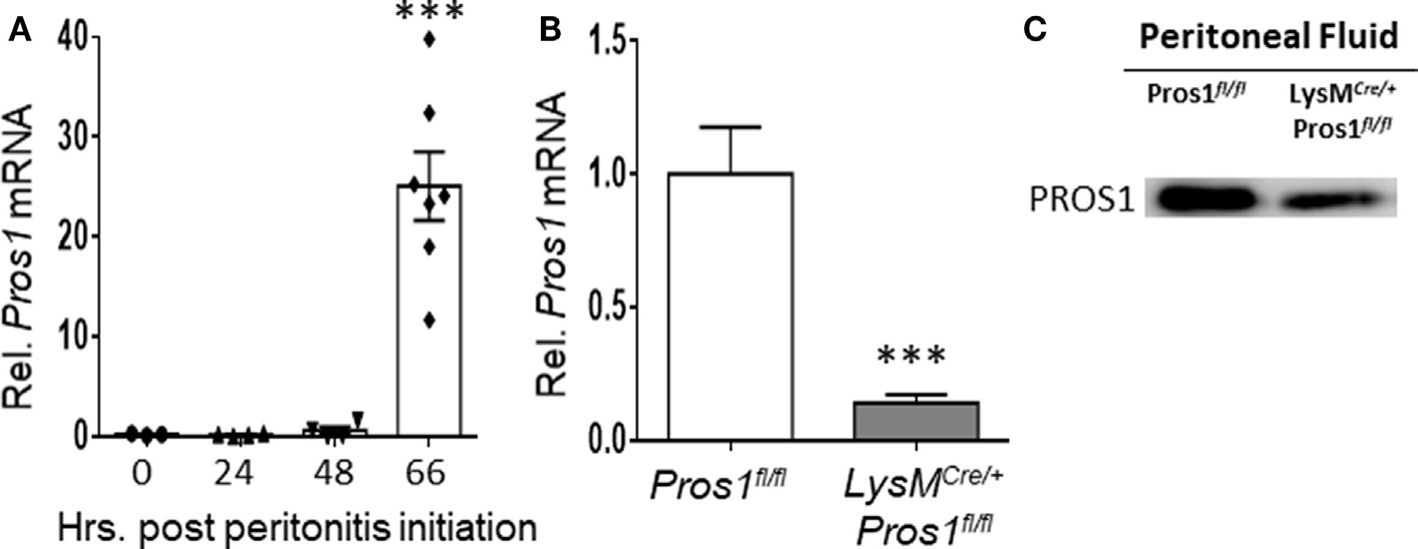
Protein S (PROS1) mRNA expression in resolution phase macrophages. **(A)** Peritoneal cells were harvested from unchallenged *Pros1^fl/fl^* mice or mice undergoing zymosan A-initiated peritonitis for 24, 48, or 66 hrs. RNA was extracted from isolated macrophages, and *Pros1* transcript levels were quantified by RT-qPCR and normalized to GAPDH (*n* = 3–7 mice per time point). Results represent the distribution of individual mice and the mean ± SEM. Statistical significance by one-way ANOVA (***P-value < 0.0001) is indicated. **(B)** PROS1 expression in *Pros1^fl/fl^* and LysM*^Cre/+^*; *Pros1^fl/fl^* resolution phase macrophages (66 h post peritonitis initiation) was quantified by RT-qPCR. Results are presented as the mean ± SEM from six mice/genotype. Student’s *t*-test, ***P ≤ 0.0001. **(C)** Peritoneal fluids from individual mice were collected 66 h after zymosan A injection and analyzed for PROS1 protein levels by Western blot. A representative blot of three experiments (three to five mice per experiment) is shown.

The phagocytosis of apoptotic neutrophils is a key step during the resolution of inflammation. To investigate the role of PROS1 during the resolution of inflammation, we utilized the zymosan A-induced peritonitis as a prototypic model (35). Peritoneal macrophages were harvested from unchallenged mice or during the inflammatory (24 h) and resolving (48–66 h) phases of peritonitis. *Pros1* mRNA levels in isolated macrophages were quantified by RT-qPCR and normalized to *Gapdh*. Our results indicate that resident macrophages (present at 0 h), inflammatory, and early resolution phase macrophages (24 h) expressed very little *Pros1*, whereas a sharp upregulation of *Pros1* mRNA in resolving macrophages (66 h) was observed (Figure 1A). To verify *Pros1* upregulation in macrophages, we measured *Pros1* transcripts in peritoneal macrophages of LysM*^Cre/+^*; *Pros1^fl/fl^* mice, in which *Pros1* expression is ablated following Cre-mediated excision in myeloid cells. We did not observe basal expression nor zymosan-induced upregulation of *Pros1* in peritoneal macrophages isolated from LysM*^Cre/+^*; *Pros1^fl/fl^* mice (36, 37) (Figure 1B). During the resolution phase, PROS1 protein levels present in the peritoneal fluids of LysM*^Cre/+^*; *Pros1^fl/fl^* mice, were also significantly reduced compared to *Pros1^fl/fl^* controls (Figure 1C). Thus, macrophages seem to express higher levels of PROS1 during the resolution of inflammation and contribute to its peritoneal levels.

### PROS1 Deficiency in Resolution Phase Macrophages Impairs Their Ability to Engulf Apoptotic Remnants

**Figure 2:**
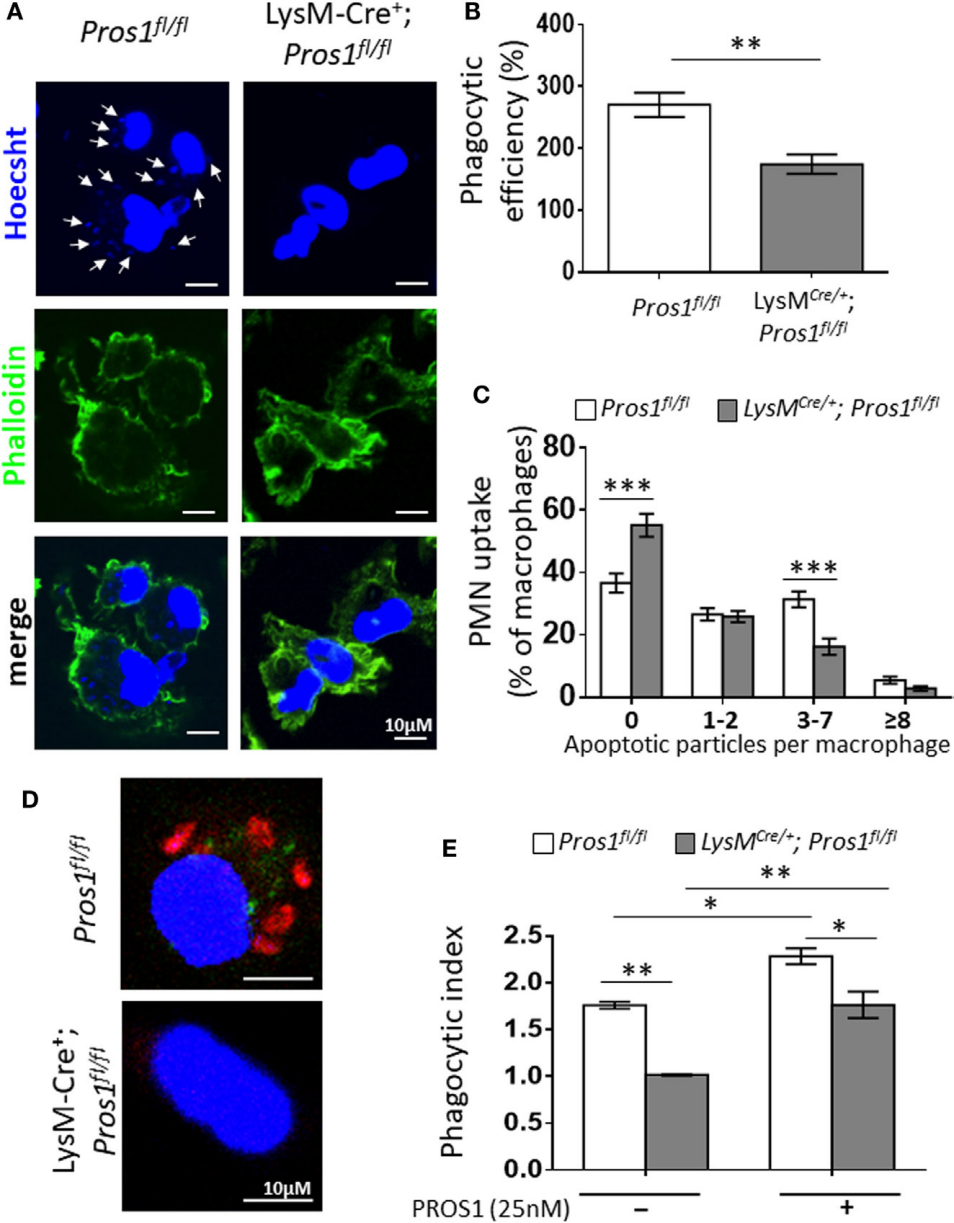
Protein S (PROS1)-deficient macrophages demonstrate a decreased engulfment of apoptotic neutrophils *in vivo*. **(A)** Resolution phase peritoneal macrophages from *Pros1^fl/fl^* (left) and LysM*^Cre/+^*; *Pros1^fl/fl^* (right) mice were isolated and stained with Hoechst (blue) and FITC-phaloidin (green). Z-stack 3D confocal images were taken with a Nikon A1-R microscope. **(B)** The number of PMN particles in each macrophage was enumerated using the Nikon NIS-Elements microscope imaging software, and the percentage of phagocytic macrophages and the number of apoptotic particles (arrows in **A**) per macrophage were calculated into the overall phagocytic efficiency index. **P = 0.004 (*t*-test). **(C)** Engulfment according to thresholds of intracellular apoptotic particles was calculated. Two-way analysis of variance (ANOVA), ***P ≤ 0.0001. **(D)** Confocal images of *in vitro* phagocytosis using pre-labeled apoptotic Jurkat cells (red). **(E)** The phagocytic index was calculated in the absence or presence of purified PROS1. Two-way ANOVA, *P ≤ 0.02, **P = 0.005. Results are representative images **(A,D)** or means ± SEM **(B,C,E)** from at least three independent experiments (*n* = 8–11 mice; over 1,500 macrophages scored).

The clearance of apoptotic neutrophils from resolving inflammation sites is essential for resolution and return to homeostasis (38). Considering the role of PROS1 in the phagocytosis of ACs (39), and its expression by resolution phase macrophages (Figure 1), we examined whether *Pros1*-deficient macrophages exert modified uptake of apoptotic neutrophils. To this end, we determined the phagocytic capacity *in vivo* of peritoneal resolution phase macrophages between LysM*^Cre/+^*; *Pros1^fl/fl^* (cKO) and their control *Pros1^fl/fl^* littermates. Our results indicate that macrophages deficient in PROS1 display a reduced ability to phagocytose apoptotic particles *in vivo* (Figures 2A–C). Quantification of this phenomenon showed that PROS1-deficient macrophages had impaired phagocytic capacity, reflected by a lower phagocytic efficiency index compared to control cells (174 ± 16 and 271 ± 20%, respectively, Figure 2B). The majority of PROS1-deficient macrophages did not engulf any apoptotic particles (55 ± 4% compared to 37 ± 3% in controls) (Figure 2C). While a similar percentage of cKO and control cells had engulfed one or two apoptotic particles (25.9 ± 2 and 26.6 ± 2%, respectively), control cells were twice as active in engulfing three to seven apoptotic moieties compared to cKO cells (31.4 ± 2.5 and 16.2 ± 2.6%, respectively). Finally, the uptake efficiency declined for both cell types scored with 8 or more Hoechst-positive foci, with a nonsignificant trend pointing to decreased efferocytosis in cKO cells, with 5.4 ± 1% of control cells, but only 2.8 ± 1% of cKO cells (Figure 2C).

We next tested whether the addition of purified PROS1 would rescue the impaired phagocytosis exhibited by PROS1-deficient macrophages. For this, we performed an *ex vivo* phagocytosis assay, assessing the efferocytosis of resolution phase peritoneal macrophages that were fed with pre-labeled apoptotic Jurkat cells (Figure 2D). Akin to their *in vivo* performance, a decreased phagocytic index was recorded for PROS1-cKO macrophages (1 ± 0.008 compared to 1.7 ± 0.03 in controls) (Figure 2E). The supplementation of exogenous PROS1 rescued the phagocytic performance of cKO cells, bringing it to normal levels of untreated control macrophages, and augmented the phagocytic capacity of *Pros1^fl/fl^* control cells (phagocytic indices of 1.7 ± 0.14 and 2.2 ± 0.08, respectively) (Figure 2E).

Taken together, our results identify endogenously expressed PROS1 as an important mediator of efferocytosis in resolution phase macrophages. Given the importance of phagocytic macrophages in clearing apoptotic neutrophils during the resolution of inflammation, we conclude that *Pros1*-deficient macrophages exhibit a hampered pro-resolving phagocytic phenotype.

### PROS1-Deficient Macrophages Display Reduced Reprogramming

**Figure 3:**
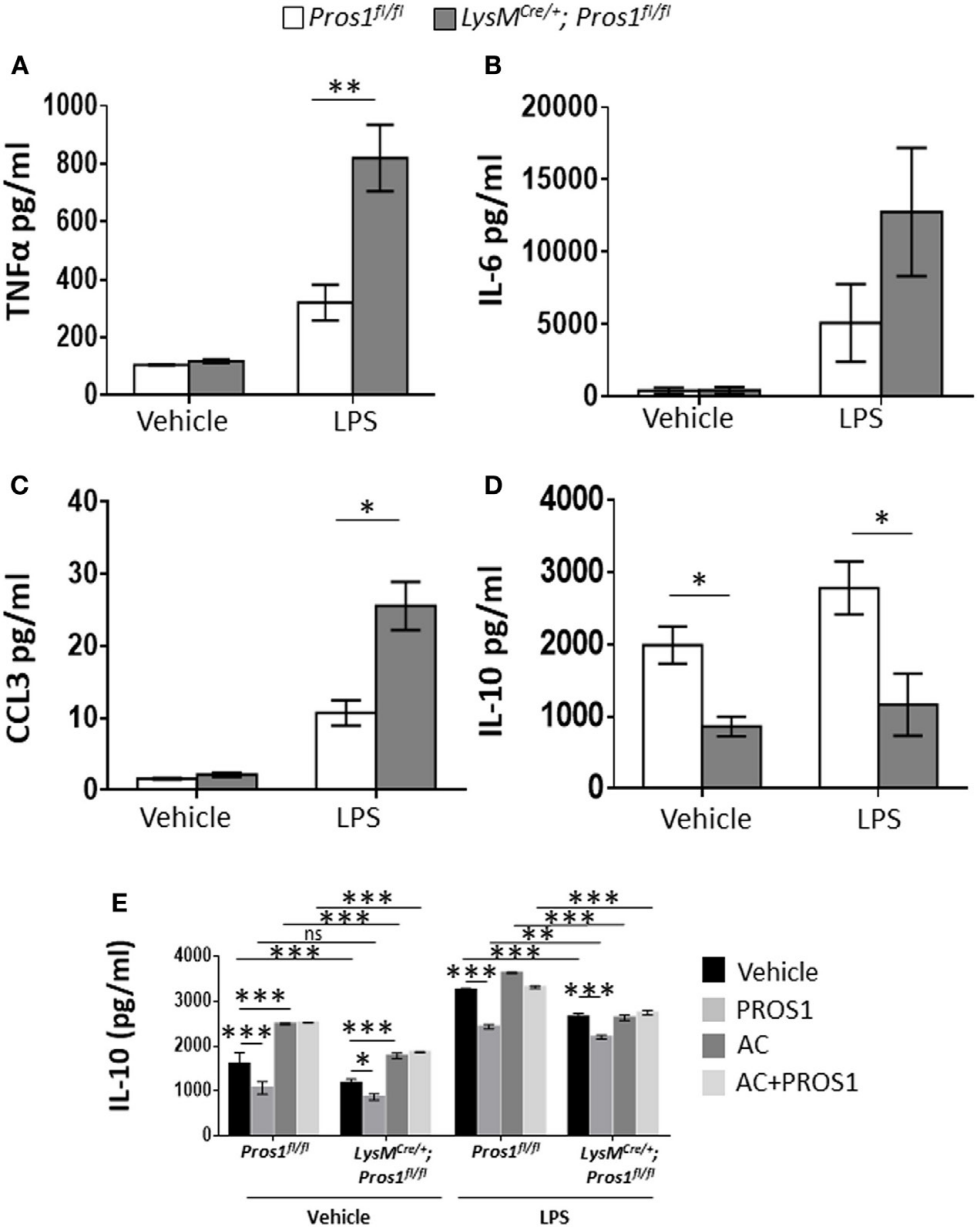
Protein S (PROS1)-deficient macrophages display reduced reprogramming. Macrophages from Pros1*^fl/fl^* or LysM*^Cre/+^*; Pros1*^fl/fl^* mice were isolated from peritoneal exudates 66 h after zymosan A injection and incubated for 24 h with vehicle or lipopolysaccharide (LPS, 1 µg/ml). Then, supernatants were collected and analyzed for their content of TNFα [**(A)**, **P = 0.003], IL-6 **(B)**, CCL3 [**(C)**, *P = 0.02], and IL-10 [**(D)**; *P ≤ 0.03] by standard ELISA. **(E)** IL-10 secretion by untreated or LPS-stimulated control and *Pros1*-cKO peritoneal macrophages, or by macrophages supplemented with either PROS1 (25 nM), apoptotic cells (AC) or both (AC + PROS1). Results represent the means ± SEM of at least three independent experiments. Two-way ANOVA, *P = 0.02, ***P ≤ 0.0001.

Apoptotic neutrophil engulfment by resolution phase macrophages results in their conversion from pro-inflammatory cells to anti-inflammatory/reparative ones (40). These M2-like macrophages prevent unwanted excessive inflammatory responses during the resolution phase of inflammation and promote tissue repair. To determine whether PROS1 expressed by resolution phase macrophages plays a role in this *in vivo* transition, termed reprogramming, we isolated resolution phase peritoneal macrophages 66 h post zymosan A treatment from *Pros1*-proficient or deficient mice. Isolated macrophages were then stimulated with LPS, and the secretion of pro-inflammatory cytokines and chemokines (TNFα, IL-6, and CCL3) or the anti-inflammatory cytokine IL-10 was determined (Figures 3A–D). Our results indicate that following LPS stimulation, PROS1-deficient macrophages secreted significantly elevated levels of TNFα in comparison to their *Pros1^fl/fl^* counterparts (820 ± 114.6 and 320.3 ± 61.3 pg/ml, respectively). The secretion of CCL3 and IL-6 was also elevated in cKO macrophages following stimulation with LPS in comparison to *Pros1^fl/fl^* ones, although the latter was not statistically significant (25.6 ± 3.3 versus 10.7 ± 1.8 pg/ml for CCL3 by cKO and controls, and 12,771 ± 4,432 versus 5,106 ± 2,684 pg/ml for IL-6 by cKO and control cells, respectively) (Figures 3B,C). Concomitantly, the secretion of IL-10 from LysM*^Cre/+^*; *Pros1^fl/fl^* macrophages was significantly lower than its secretion by *Pros1^fl/fl^* macrophages under baseline conditions and upon stimulation with LPS (861 ± 136 and 1,990 ± 258 pg/ml for unstimulated cKOs and controls, and 1,165.5 ± 431 and 2,783.2 ± 368 pg/ml for stimulated cKO and controls, respectively) (Figure 3D). Thus, *Pros1*-deficient macrophages present a shift toward exacerbated pro-inflammatory cytokine secretion, suggesting hampered reprogramming compared to their control counterparts. Since IL-10 is a key cytokine in macrophage reprogramming (5), we next determined the effect of exogenous PROS1 on AC-induced IL-10 secretion *ex vivo*. Our results indicate that ACs promoted the secretion of IL-10 in both control and *Pros1*-cKO mice when unstimulated, but upon LPS stimulation, this effect was absent in PROS1-deficient macrophages (Figure 3E). Unexpectedly, the addition of PROS1 was unable to enhance the effect of ACs on IL-10 secretion in PROS1-deficient mice and in fact reduced IL-10 secretion from untreated and LPS-stimulated macrophages (both WT and PROS1-deficient). This may be due to the biochemical nature of PROS1. PROS1 acts as a bridging molecule that binds phosphatidylserine (PstSer) exposed on the outer leaflets of ACs *via* its amino terminus and to the extracellular domain of TAM receptors on phagocytes and macrophages *via* its carboxy terminus (24). Thus, the addition of excess PROS1 to the combined culture of macrophages and ACs may saturate the binding sites on ACs and macrophages without physically bridging between them. It is conceivable that the controlled and sequential addition of PROS1 to macrophages and ACs would favor the bridging and subsequent reduction of IL-10 production. Nevertheless, our results indicate that compared to controls, PROS1-deficient macrophages present a hampered response to AC uptake that results in a pro-inflammatory imbalance with elevated TNFα and CCL3 as well as lower IL-10 production and could only partially be rescued by exogenous PROS1.

### *Pros1*-Deficient Macrophages Express Reduced Levels of Pro-Resolving Enzymes

**Figure 4:**
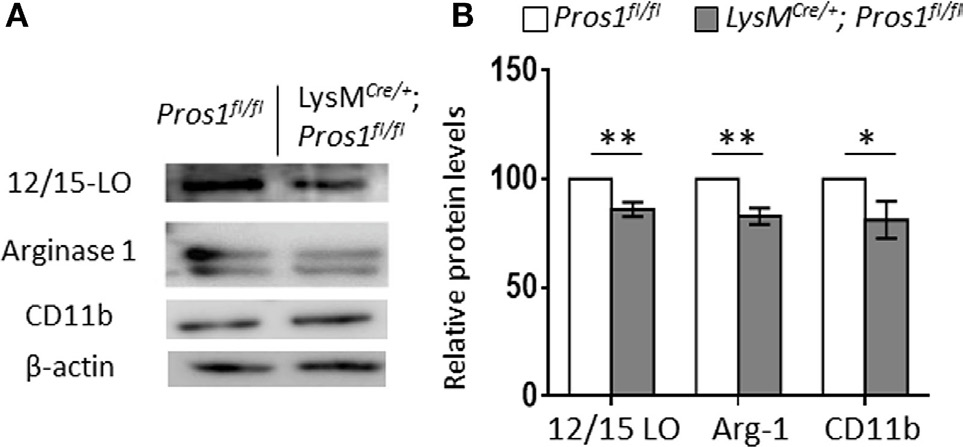
Protein S (PROS1)-deficient macrophages express reduced levels of resolution phase enzymes. Peritoneal macrophages from Pros1*^fl/fl^* or LysM*^Cre/+^*; Pros1*^fl/fl^* mice (*n* = 5/group) were isolated 66 h after zymosan A injection and lysed, and their protein content was analyzed by Western blot for the resolution phase markers 12/15-LO, arginase-1, and CD11b. A representative blot is shown **(A)**. Band intensities were quantified, and graphs represent the normalized means ± SEM from three independent experiments **(B)**. Student’s *t*-test, *P = 0.03, **P ≤ 0.02.

During the resolution of inflammation, the engulfment of apoptotic neutrophils leads to macrophage metamorphosis from an M1-like phenotype to an M2-like phenotype and then to a pro-resolving phenotype (Mres) highlighted by the expression of the functionally important enzyme 12/15-LO (40). These changes are associated with reprogramming of the engulfing macrophages (41). Since our results so far indicate that *Pros1* expression by resolution phase macrophages is required for their uptake of apoptotic neutrophils and reprogramming, we sought to analyze the expression of proteins that are instrumental to inflammation and its resolution (19, 42–44). 12/15-LO is a key enzyme involved in the synthesis of lipoxins, protectins, resolvins and other specialized lipid mediators that promote the resolution of inflammation by macrophages (45–47). Arginase-1 enzymatically inhibits nitric oxide (NO) production by inducible NO synthase (iNOS), thereby supporting an anti-inflammatory/reparative milieu (48). Since iNOS characterizes M1-like macrophages and is highly expressed by pro-inflammatory macrophages, the levels of arginase-1 expression reflect the maturation level of macrophages and their progression to the reparative phenotype during resolution (40). To evaluate macrophage maturation and differentiation, peritoneal macrophages from LysM*^Cre/+^*; Pros1*^fl/fl^*; and Pros1*^fl/fl^* mice were isolated 66 h post peritonitis initiation, and their protein content was immuno-blotted for the macrophage M2/maturation markers arginase-1 and CD11b as well as for the pro-resolving enzyme 12/15-LO (Figure 4). Our results indicate that LysM*^Cre/+^*; *Pros1^fl/fl^* macrophages express significantly lower levels of all three proteins (86, 83, and 81% of controls, for 12/15-LO, arginase-1, and CD11b, respectively). Thus, LysM*^Cre/+^*; *Pros1^fl/fl^* macrophages display a reduced capacity to mature and convert to the M2-like and Mres phenotypes during the resolution of inflammation. 12/15-LO catalyzes the derivation of DHA into resolvin (Rv) D1. Given the importance of RvD1 as a key mediator involved in the resolution of inflammation, we tested whether the decreased levels of 12/15-LO in PROS1-deficient macrophages (Figure 4) would also affect the potential production of RvD1 by these cells. We found a 25% decrease in the production of RvD1 by PROS1-defiecient cells compared to that of controls (1,899 ± 33.2 and 2,527 ± 57.8 pg/ml for cKO and controls, respectively) (Figure S1 in Supplementary Material). Thus, the reduced expression of 12/15-LO in PROS1-deficient resolution phase macrophages results in a reduced capacity to produce specialized pro-resolving lipid mediators. Taken together, our results indicate that macrophage-derived PROS1 is a molecular effector in the uptake of apoptotic neutrophils and participates in the consequent reprogramming and maturation of macrophages in resolving inflammation.

## Discussion

Protein S is a pleiotropic mediator involved in various processes, such as vasculogenesis, blood clotting, and immune regulation (29, 33, 37, 49). While hepatocytes are considered to be the major source of plasma PROS1, significant contributions to the volume and function of PROS1 were attributed to other sources, such as endothelial and T cells (29, 33, 37). Our findings now indicate that PROS1 is produced by macrophages during the resolution of murine peritonitis, and consequently its production and release by peritoneal macrophages as well as other cells contribute to peritoneal levels of PROS1 (Figure 1). While peritoneal PROS1 levels probably increase during the inflammatory phase of peritonitis, due to exudation of plasma proteins, it is unlikely that peritoneal PROS1 will remain at high levels in the absence of additional sources. Along these lines, PROS1 from macrophages seems to be an important component in its peritoneal levels during resolution. Notably, PROS1 produced by resolution phase macrophages is functionally important. This could be due to a critical concentration of the peritoneal protein or due to a local regulation of PROS1 function as seen in the lymph nodes (29) and the retina (33). Similarly, our results indicate that the discrete secretion of macrophage-generated PROS1 is essential for key-resolving features of efferocytosis and molecular reprogramming. Such localized secretion could be envisioned in the contact area between apoptotic PMN and the macrophages that engulf them (50).

Protein S was previously found to bind the tyrosine kinase receptors from the TAM family and mediate their interactions with ACs through the binding of PstSer (39, 51–53). As expected in this setting, the deficiency in PROS1 production by efferocytosis-competent macrophages resulted in a significant reduction in the uptake of apoptotic PMNs in LysM*^Cre/+^*; *Pros1^fl/fl^* macrophages, which was rescued following the addition of purified PROS1 (Figure 2). The engulfment of apoptotic PMNs is essential for the resolution of inflammation [reviewed in Refs. (5, 40)]. This is in part due to the phenotypic changes that take place in the engulfing monocytes/macrophages (5, 10, 54). These changes, also termed macrophage reprogramming/immune-silencing, are characterized by a reduction in the production of pro-inflammatory cytokines and chemokines concomitant with an increase in the production of anti-inflammatory cytokines, such as IL-10, upon exposure to bacterial moieties (21, 41, 55). Notably, the TAM receptors were previously shown to block TNFα- and TLR-mediated inflammatory signals (25, 31, 56). In addition, the PROS1-mediated phagocytosis of ACs by peripheral blood monocytes contributes to the elimination of dying and dead cells in the circulation (39), thus avoiding the induction of harmful inflammatory responses. Our current results indicate that a myeloid-specific deficiency in *Pros1* culminates in increased amounts of the pro-inflammatory mediators TNFα and CCL3 and reduced amounts of the anti-inflammatory/pro-resolving cytokine IL-10, upon stimulation with LPS (Figure 3), a profile resembling pro-inflammatory rather than resolution phase macrophages.

During murine peritonitis, CD11b^med^ monocytes differentiate to CD11b^high^ macrophages that engulf apoptotic PMNs in a self-limiting fashion and convert to CD11b^low^-satiated macrophages (21, 35). These phenotypic changes are associated with a temporal increase in the expression of arginase-1, a hallmark of M2 macrophages that is induced by AC uptake (19, 57). 12/15-LO, an enzyme that is involved in the production of pro-resolving lipid mediators including RvD1 (1, 58) and the uptake of ACs (59), is progressively upregulated by macrophages during the resolution of inflammation. Moreover, it is a hallmark of macrophage conversion from the CD11b^high^ to the CD11b^low^ phenotype (21). Although CD11b protein levels were reduced in *Pros1*-cKO-resolving macrophages, the expression of arginase-1 and 12/15-LO—two *bona fide* markers of resolution phase macrophages—was lower in efferocytosing macrophages lacking PROS1 (Figure 4). In line with the reduced levels of 12/15-LO, less RvD1 is produced by macrophages devoid of PROS1 (Figure S1 in Supplementary Material). Together, with their impaired efferocytosis and a pro-inflammatory cytokine profile, these results suggest that the conversion to resolution phase macrophages in the absence of PROS1 is either incomplete or hampered. Hence, PROS1 production by local macrophages seems to be important in their reprogramming during the resolution phase of inflammation.

In sum, our results indicate that *Pros1* production and action in pro-resolving macrophages are key events in the termination of inflammation. PROS1 acts in various modes as it regulates both the uptake of apoptotic PMNs and the consequent reprogramming of macrophages. These findings suggest that PROS1 might be harnessed as a new strategy for the treatment of inflammatory and autoimmune disorders.

## Ethics Statement

All animal experiments were approved by the Hebrew University – Hadassah ethics committee.

## Author Contributions

DL, TB-C, and AA designed the research. DL, SS, AM, and SS-Z performed experiments, analyzed, and visualized data. TB-C and AA supervised the project. DL, TB-C, and AA wrote the manuscript.

## Conflict of Interest Statement

The authors declare that the research was conducted in the absence of any commercial or financial relationships that could be construed as a potential conflict of interest.
