# Corrigendum: Macrophage-Derived Protein S Facilitates Apoptotic Polymorphonuclear Cell Clearance by Resolution Phase Macrophages and Supports Their Reprogramming

**DOI:** 10.3389/fimmu.2020.412281

**Published:** 2020-11-30

**Authors:** Delphine Lumbroso, Soaad Soboh, Avi Maimon, Sagie Schif-Zuck, Amiram Ariel, Tal Burstyn-Cohen

**Affiliations:** ^1^ Department of Biology, The Faculty of Natural Sciences, University of Haifa, Haifa, Israel; ^2^ Faculty of Dental Medicine, Institute for Dental Sciences, Hadassah Medical School, Hebrew University of Jerusalem, Jerusalem, Israel

**Keywords:** inflammation, macrophages, protein S, PROS1, apoptosis, efferocytosis

In the published article, there was an error in affiliation 1. Instead of “Faculty of Dental Medicine, Institute for Dental Sciences, Hadassah Medical School, Hebrew University of Jerusalem, Jerusalem, Israel,” it should be “Department of Biology, The Faculty of Natural Sciences, University of Haifa, Haifa, Israel.”

In the published article, there was an error in affiliation 2. Instead of “Department of Biology, The Faculty of Natural Sciences, University of Haifa, Haifa, Israel,” it should be “Faculty of Dental Medicine, Institute for Dental Sciences, Hadassah Medical School, Hebrew University of Jerusalem, Jerusalem, Israel.”

The authors apologize for these errors and state that this does not change the scientific conclusions of the article in any way. The original article has been updated.

