# Supplementary figures and images for: Macrophage-Derived Protein S Facilitates Apoptotic Polymorphonuclear Cell Clearance by Resolution Phase Macrophages and Supports Their Reprogramming
Source: Front Immunol. 2018 Mar 1;9:358. doi: 10.3389/fimmu.2018.00358 (PMC5837975; doi:10.3389/fimmu.2018.00358)

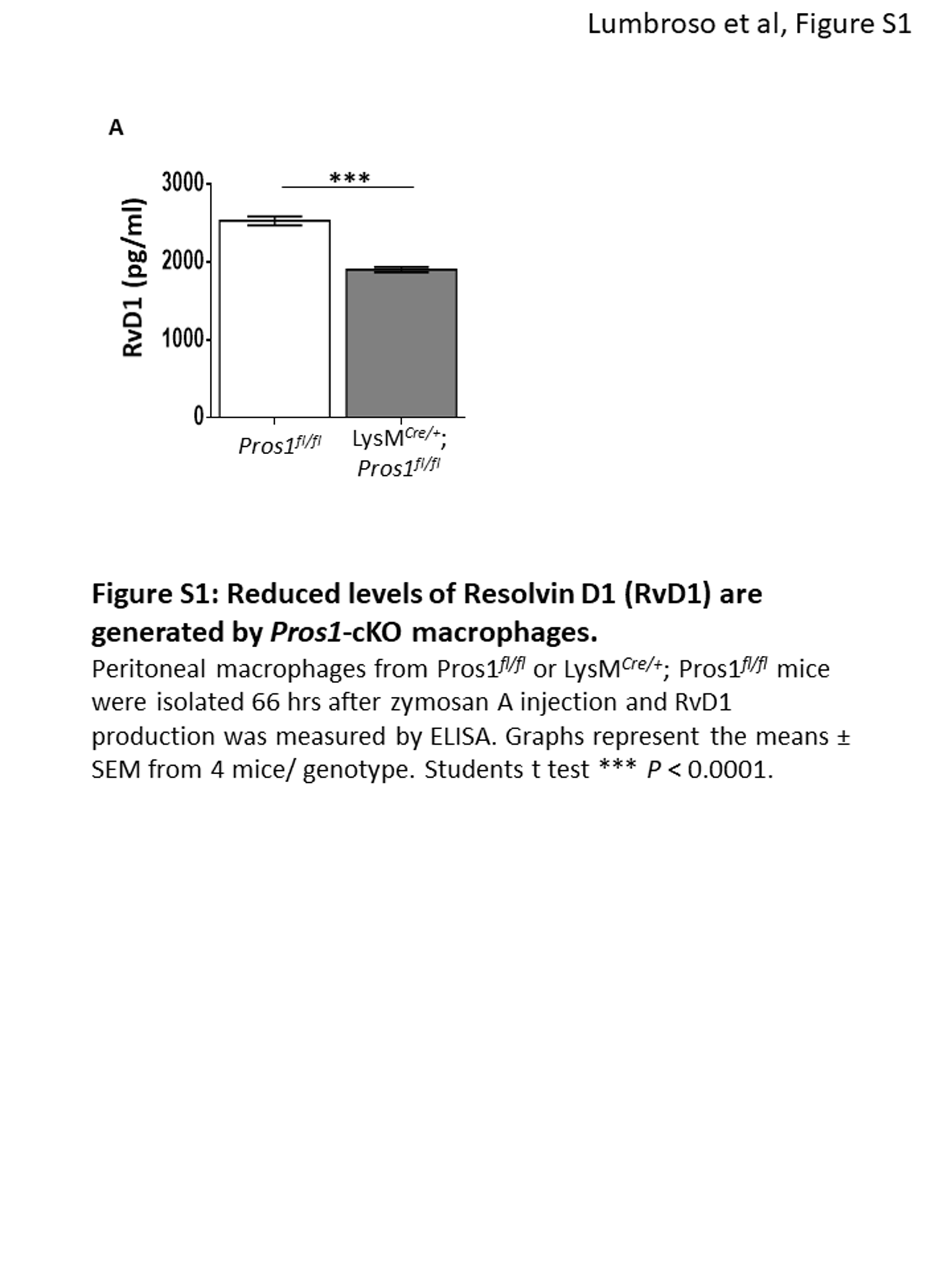

Supplement: Supplementary file 1 [file image_1.tif]
